# Contactless in vitro detection of carboxyhemoglobin using hyperspectral imaging (HSI)

**DOI:** 10.1007/s12024-025-00949-1

**Published:** 2025-02-04

**Authors:** P. Czarkowski, C. Babian, St Lüdtke, S. Baumann, J. Dreßler

**Affiliations:** 1 Institut für Rechtsmedizin, Medizinische Fakultät, Universität Leipzig, Johannisallee 28, 04103 Leipzig, Germany; 2 Institute of Visual & Analytic Computing, University of Rostock, Abert-Einstein-Straße 21, 18059 Rostock, Germany

**Keywords:** Hyperspectral imaging, Carboxyhemoglobin, Forensic medicine, Forensic toxicology, CO-Hb

## Abstract

Hyperspectral imaging (HSI) allows for the contactless analysis of the composition of substances based on the reflected light and is already used in various areas of medicine. The carboxyhemoglobin (CO-Hb) concentration in blood of suspected fire victims serves to prove vitality and the cause of death. However, this metric is usually determined by spectrophotometry in the laboratory. The present study provides the basis for the future development of methods for determining CO-Hb concentrations right at the scene of a corpse or at necropsy using mobile HSI. Human erythrocyte concentrate was mixed with gaseous carbon monoxide using an aerator to produce a series of samples, which were analyzed for their CO-Hb concentration (2.9; 9.7; 18; 27.9; 39.9; 51.9; 62.3; 73.4% CO-Hb) using established spectrophotometric blood gas analysis. These blood samples were stored in a cool place at 4 °C, dripped onto a spot plate every 7 days over a period of 6 weeks, and photographed under standardized conditions (ambient lighting, distance and angle of the camera to the sample, camera settings) using the HSI camera SPECIM IQ. This device analyzes each image in the wavelength range from 400 to 1000 nm in 204 spectral bands. The data sets were used to train a lasso regression model, which provides predicted values for the CO-Hb concentration of the blood sample based on their hyperspectral properties. The results were then compared with the results of spectrophotometric measurements. The lasso regression model allowed the prediction of the CO-Hb concentration of the samples with a mean prediction error of 4.46 percentage points, independent of the sample age. Further investigations regarding pre-analytical influencing factors such as variable ambient light and tissue scattering effects, are planned to validate the robustness of the method and realize practical implementations.

## Introduction

Although the total number of cases of carbon monoxide (CO) poisoning has been falling steadily since 2012, deaths have remained at a consistently elevated level since 1998 and are among the most common fatal cases of poisoning. In 2021, 397 people died from carbon monoxide poisoning in Germany. [5]

CO is a tasteless, odorless, and invisible gas. It is produced whenever organic materials are burned with an inadequate supply of oxygen necessary to produce complete combustion. For example when using portable charcoal barbeques in enclosed spaces relevant amounts of CO can accumulate and recent years have shown some increased popularity and consecutive rising of both accidents and suicides related to those [6]. Forensic analysis of CO-Hb concentration is one of the most important vital signs in suspected burn victims, since only a breathing person who has inhaled the gas would show elevated CO-Hb levels.

A variety of laboratory analytical methods are currently available for determining the concentration of carbon monoxide in blood. Many types of these are based on the different spectral properties of hemoglobin, depending on its bound molecules. Other methods use gas chromatography to quantify the amount of released CO from the sample. Due to low cost of the methods, its broad availability and ease of use, the spectrometric blood gas analysis is considered the “gold-standard” for determining the concentration of carbon monoxide in blood in the laboratory, although the results can be influenced, for example when optical impairments of the samples occur (see [18, 20, 21]). To date, there are no established methods for the immediate quantitative determination of the CO-Hb concentration in preclinically deceased persons (e.g. at the site of the body or the first necropsy) [9]. Features such as bright red *livor mortis*, violet-colored nail beds, and “cherry-red” blood may indicate carbon monoxide poisoning. Nevertheless, these indications are highly dependent both on the examiner’s experience and judgement and could also have been caused by postmortem exposure to cold [7].

Hyperspectral imaging (HSI) enables examinees to objectively determine the visible and near-infrared (vis–NIR) spectrum of samples on site, regardless of their experience, unfavorable environmental factors, or the availability of stationary, off-site laboratory equipment [14].

In the present study, the determination of carbon monoxide concentration in human blood was investigated using a mobile HSI camera to analyze the reflectance spectrum of artificially saturated samples with CO.

We wanted to examine the fundamental feasibility of the determination of the CO Hb concentration based on the optical properties of a sample recorded with a hyperspectral camera. Therefore, the following questions were investigated:

1) Given the acquisition of high-quality data, can HSI in a standardized setting (which still could be achieved in a crime scene) assess the COHb concentration in human blood quantitatively?

2) Is the HSI predicted COHB value stable over a period of 6 weeks?

## State of research

The red color of blood is caused by light absorption of heme in hemoglobin. The porphyrin ring of heme has an extended system of conjugated double bonds. This delocalized π-electron system absorbs light in the visible wavelength range and is sensitive to the change in the chemical environment. Consequently, the degree of oxygenation of hemoglobin affects the hue of blood and different oxygenation states of hemoglobin can be clearly distinguished by their respective absorption spectra. All hemoglobin spectra are dominated by an intense light absorption in the range of 400–430 nm (Soret band). In addition, oxygenated hemoglobin shows two absorption bands at 576 and 540 nm. De-oxygenated hemoglobin shows only one absorption band at 555 nm in this range. The absorption spectrum is similar to that of carbon monoxide-loaded hemoglobin (HbCO) [17].

When inhaled, two molecules of CO are bound to the central, positively charged iron ion (Fe2 +) of the heme group in the hemoglobin molecule (the “oxygen binding site”) with an affinity about 300 times of that of oxygen. This causes the Hb to switch to the relaxed state, which increases the affinity of Hb for oxygen. [10] This results in hypoxic symptoms, ranging from light dizziness to severe brain damage and even death. Although it seems that there is no direct correlation between concentration levels and symptoms [8], 30% CO-Hb or more is usually found in the heart blood of deathly CO poisoning cases. [13]

Classical, digital color photography records the three brightness values (intensity) for the colors red, green and blue for each image point (pixel). By comparison, hyperspectral analysis can record hundreds of intensity values per pixel in the vis–NIR spectral range, typically from 400 to 1000 nm. The Specim IQ® portable HSI camera can take said images at any location and is therefore independent of a laboratory or examination room.

The acquisition of hyperspectral images for qualitative analysis of human blood has already been investigated [4] and alternative possibilities for CO-Hb determination based on changes in the spectral properties of the various hemoglobin derivatives are also under discussion. Some studies [1, 11, 12, 16] have already tried different HSI devices to determine either the presence or quantity of carboxyhemoglobin in blood based on its spectrophotometric characteristics. Next to restricted availability and expensive laboratory work force and devices, in those methods a processing time of several hours was common.

To our knowledge, studies on the determination of CO-Hb concentration using the SpecimlQ portable HSI (hyperspectral imaging) camera have not yet been performed.

## Methods

The study was conducted at the Institute of Forensic Medicine at the University of Leipzig with the approval of the Ethics Committee (date:31.08.2021, decision no.:397/21-ck).

Anonymized, leukocyte-depleted human erythrocyte concentrates (EC’s) from donors of the Institute of Transfusion Medicine at Leipzig University Hospital were used as sample material. After completion of the examination, the remains of the EC’s were disposed of at the Institute of Forensic Medicine.

To prepare a highly saturated positive sample, a constant flow of gaseous carbon monoxide from a cartridge was introduced into 250 ml of red blood cell concentrate with a physiological concentration of 2.9% COHb for 5 min in a closed fume cupboard. This was then diluted using the untreated volume from the same EC to produce seven batches with a CO concentration of 9.7, 18, 27.9, 39.9, 51.9, 62.3, and 73.4% CO-Hb respectively. The concentrations were verified using spectrometric blood gas analysis in the laboratory.

Every 7 days over a period of 6 weeks, 100 µL of each of the samples including the control sample were dripped onto a white porcelain plate. The reflectance spectra of these eight samples were then measured using a hyperspectral camera. The samples were stored in a refrigerator at 4 °C between experiments.

**Fig. 1:**
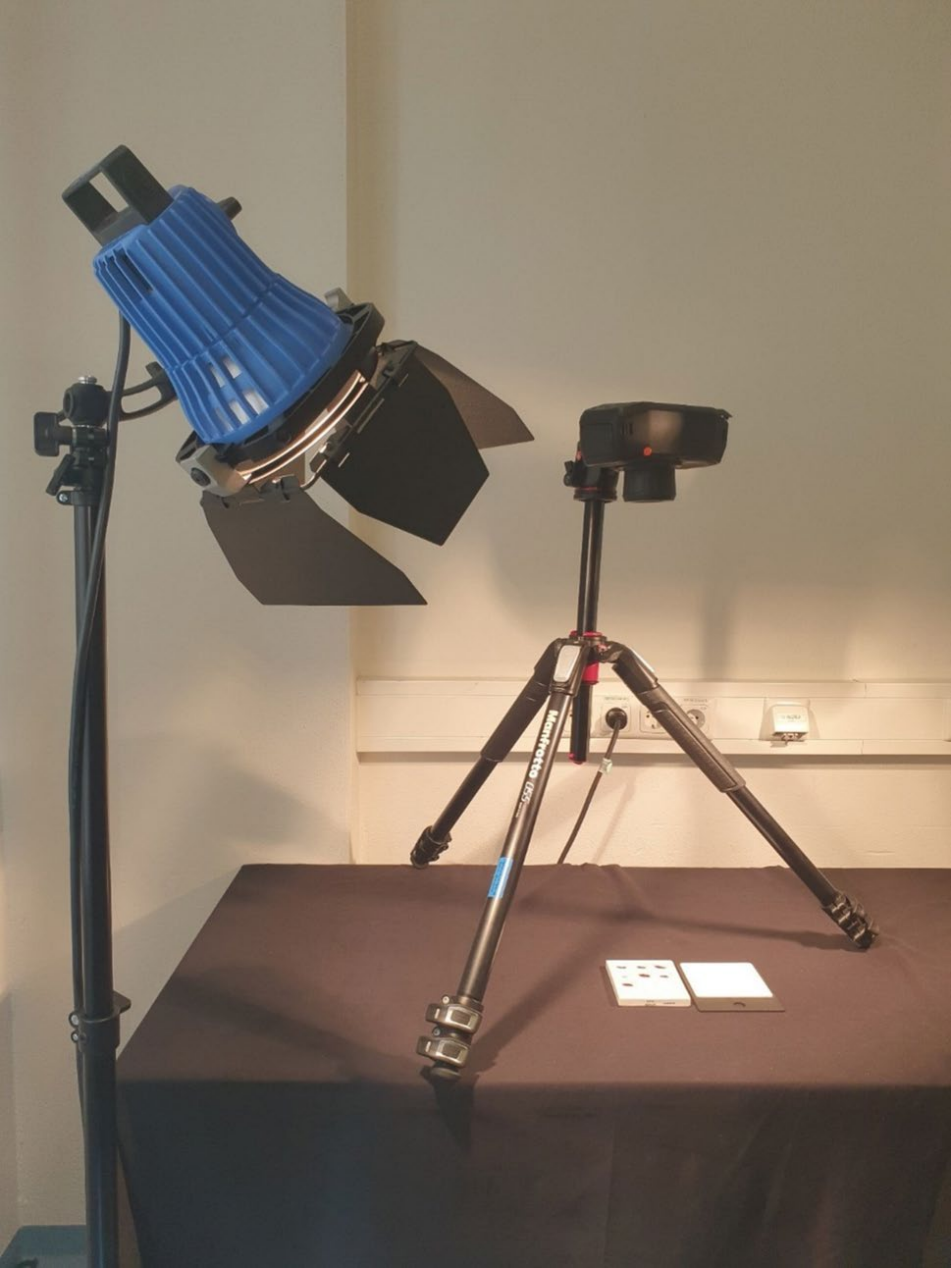
Setup: Specim IQ camera positioned above sample tile with white reference plate to the right and illuminated by halogen light

The recording conditions were standardized: Two full spectrum light sources (“ARRILITE 750 Plus” halogen lamps) illuminated the sample field from both sides from approximately 120 cm, which was located vertically under the lens of the camera on a tripod (Fig. 1 – Setup).

The Specim IQ® hyperspectral camera (Specim, Spectral Imaging Ltd., Oulu, Finland), weighing 1.3 kg and measuring 21 × 9x7 cm, is a portable system for recording the intensity of light in the wavelength range from 400 to 1000 nm in 7 nm steps. Images were taken with different exposure times (integration time) from 1 to 100 ms and 40 and 60 cm lens distance to the samples, whereby the simultaneous white balance was conducted with the aid of a reference template located in the image section. An initial analysis showed little sensitivity of recorded intensities with respect to integration time and distance to samples. Thus, an exposure time of 5 ms and 40 cm distance were used as consistent settings for all further analyses.

**Fig. 2:**
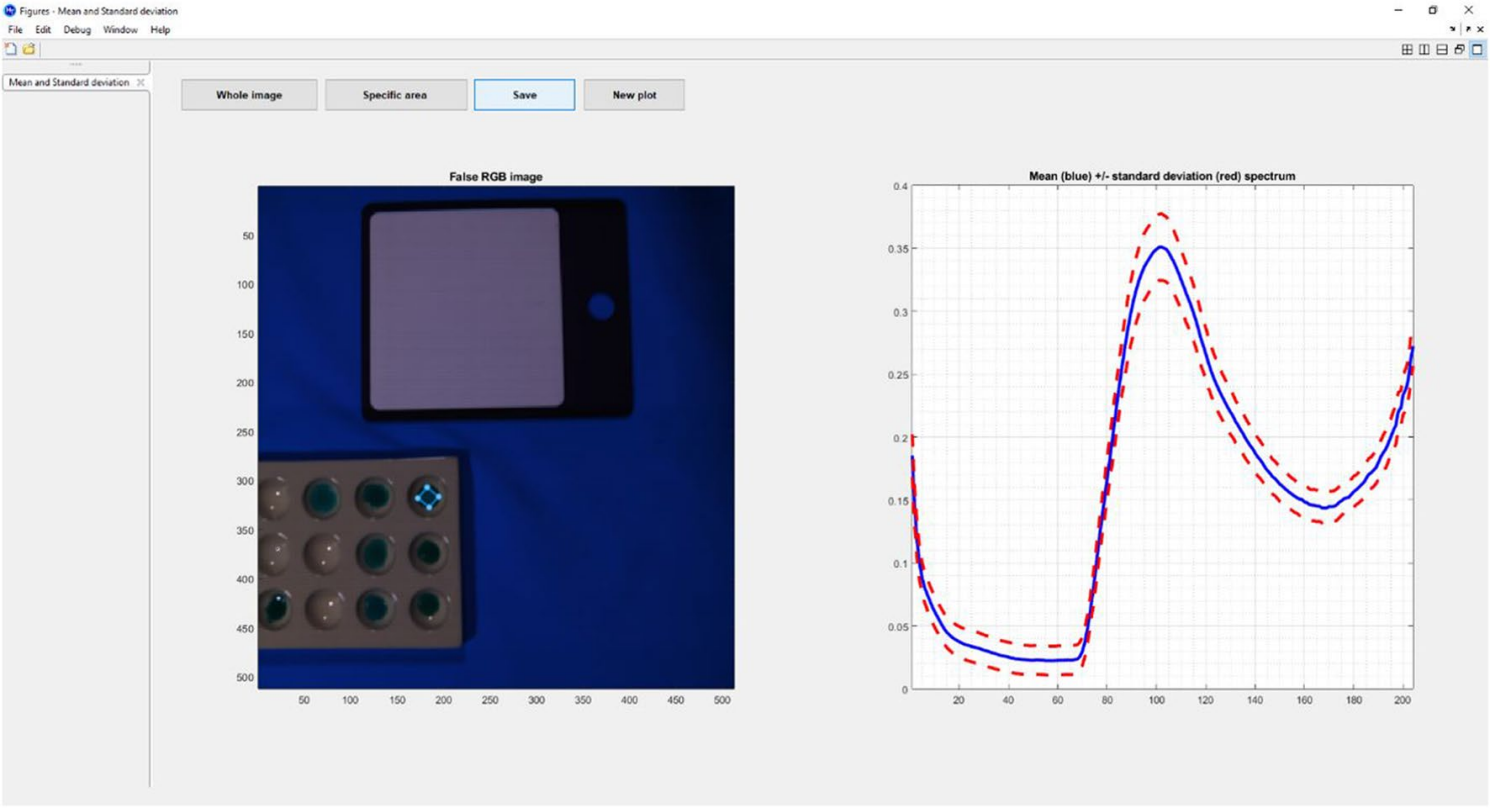
MATLAB hypertools3 Screenshot showing extracted spectra from region of interest

Each hyperspectral image contained 204 intensity values per pixel in the captured wavelength range. Therefore, the MATLAB® library HYPER-Tools was used to define an approximately 10 × 10 pixel polygon for each sample which contained a representative area of the droplet (Fig. 2—MATLAB hypertools3 Screenshot). The spectral profile of each sample was calculated as the mean of the intensities within the polygon per wavelength range.

To determine whether the recorded spectrum allowed prediction of CO-Hb concentration, a linear regression model with lasso regularization was fitted, using the intensity values of all 204 wavelength ranges as independent variables and the known CO-Hb concentration as the dependent variable. The R programming language and the “glmnet” and “ggplot2” packages were used for this statistical analysis.

The prediction error of the regression model was determined using k-fold cross-validation. The data was first divided into k = 6 subsets, whereby a subset consisted of all data (i.e. intensity values) of a given recording time point. K evaluations were performed and in the evaluation i, the data of subset i was used to determine the root mean squared error (RMSE), while all other data was used to train the model. Finally, the mean RMSE of the evaluations was reported. This procedure provides a robust estimate of the error produced by the model on new data not used in training. For the lasso regularization parameter lambda, the values 10^×^ for x ∈ {1;0.9; …;−4} were systematically tested, and the model with the lowest RMSE (determined by cross-validation) was used.

## Results

**Fig. 3:**
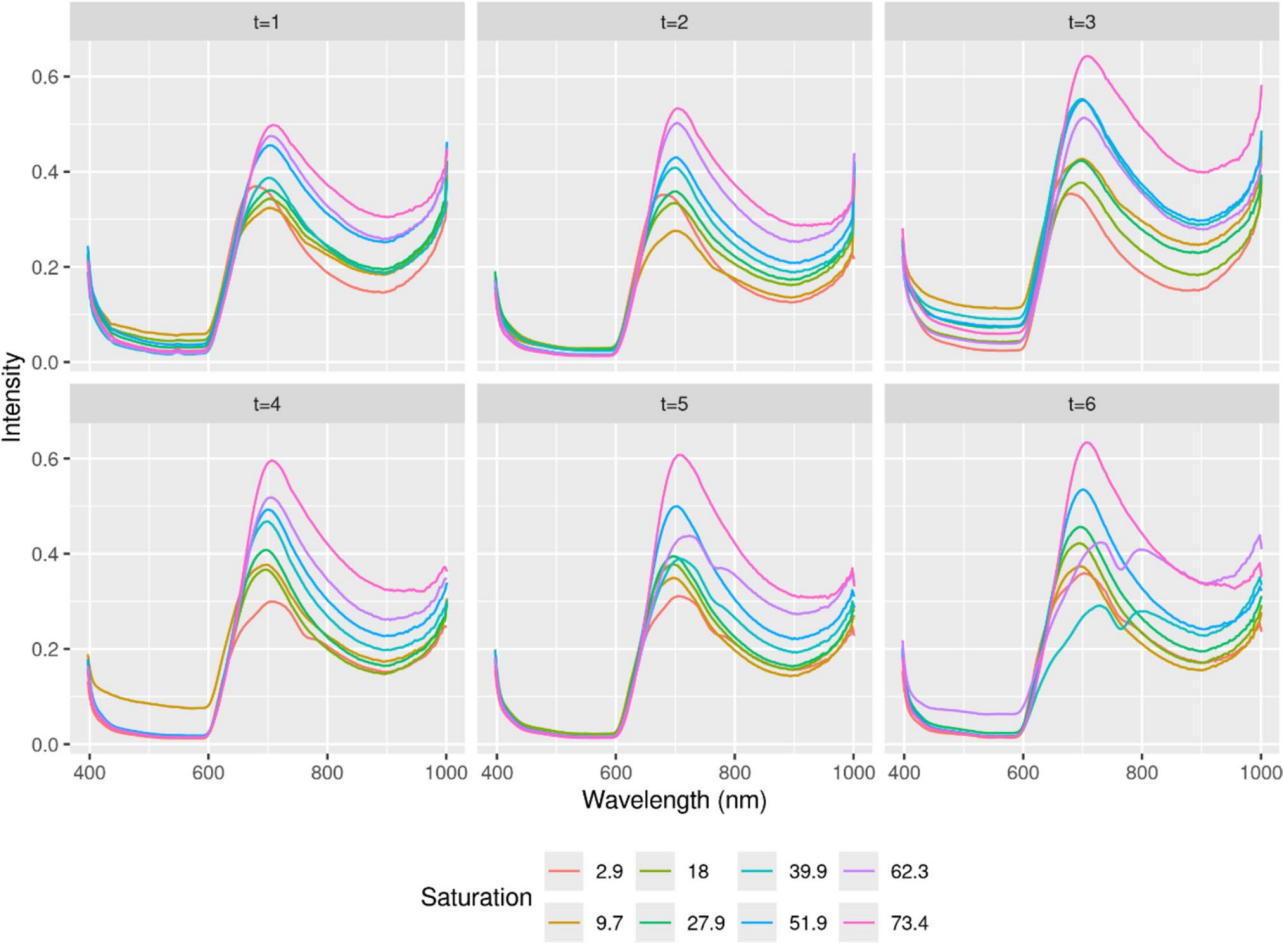
Intensity per Wavelength during six weeks

The maximum intensity is reached in frequency band 105, which corresponds to a wavelength of 702.58 nm. In weeks 5 and 6, artifacts are visible in the samples with 40% and 60% CO-Hb concentration and their spectra differ significantly from the other spectra (Fig. 3—Intensity per Wavelength during six weeks).

**Fig. 4:**
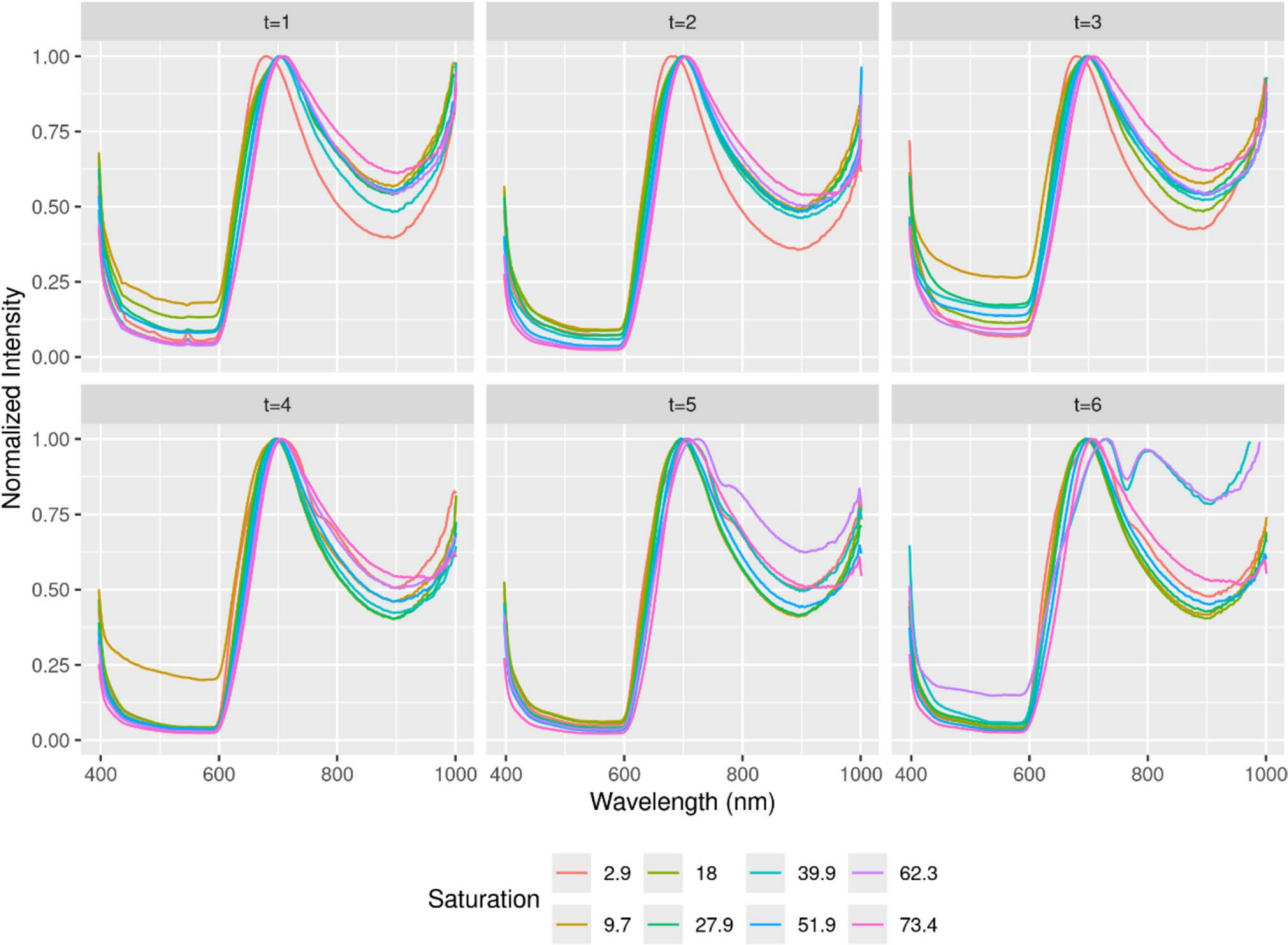
Intensity per Wavelength during six weeks (normalized)

To remove the effect of ambient light from the data and to increase the generalization capability and thus the quality of the prediction model, the intensity values of each blood drop were normalized, i.e. each intensity value was divided by the maximum intensity value of each sample (Fig. 4—Intensity per Wavelength during six weeks (normalized)).

**Fig. 5:**
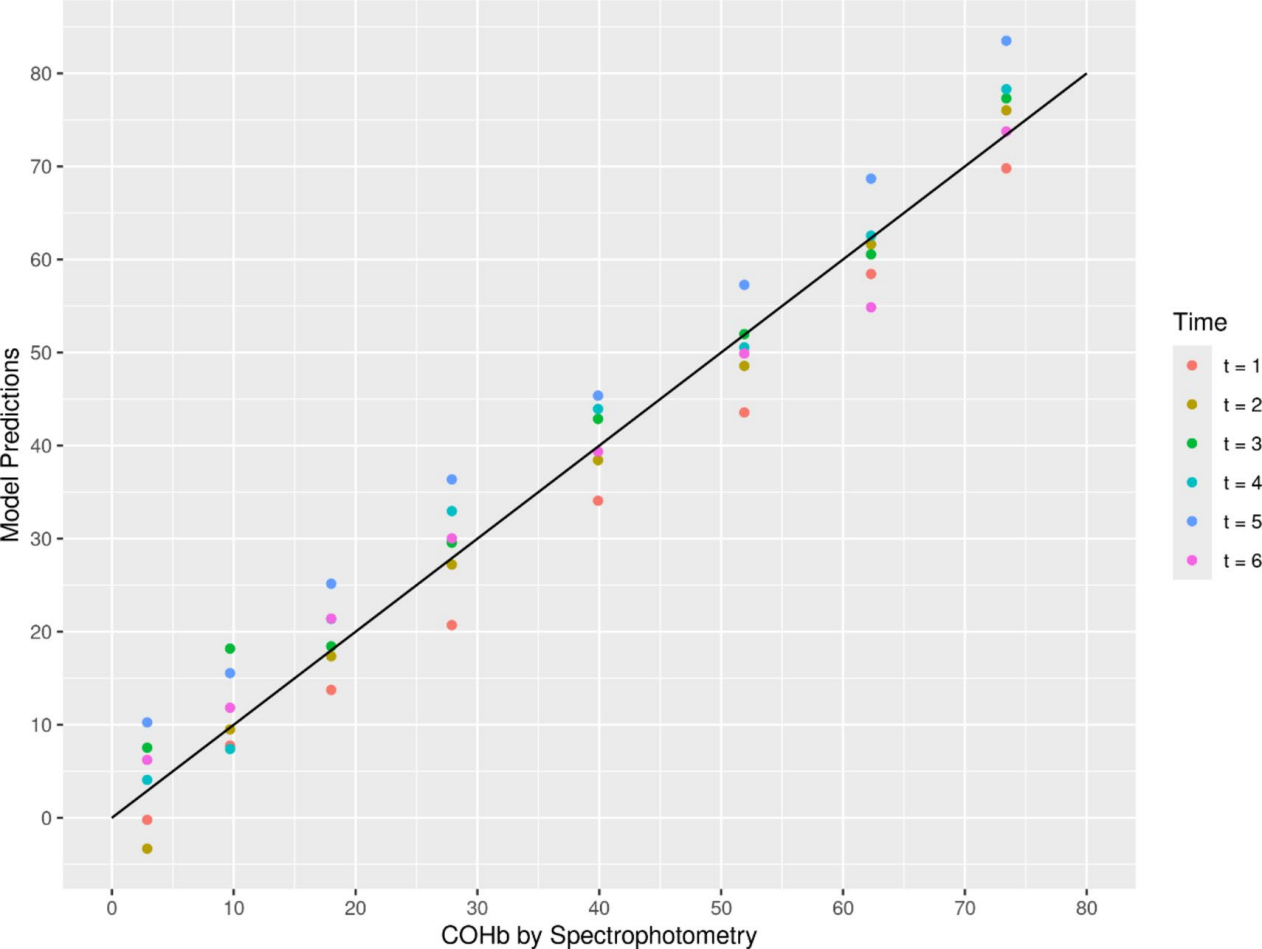
Prediction of COHb concentration by the model compared to from Spectrophotometry, week of capture in color

The lasso regression of the normalized spectra (i.e., the 204 intensity values) on the CO-Hb concentration achieved an RMSE of 4.46 percentage points determined by cross-validation, for lambda = 10^–1.5^. This means that on new data (collected with the same experimental setup) the CO-Hb concentration can be calculated with an average prediction error of approx. 4.5 percentage points. Figure 5 shows the model predictions as a function of the true CO-Hb concentration, with different colors indicating different recording times in the course of the experiment. While the prediction error varies between samples, the model prediction is homoscedastic (the variance of the errors does not depend on the true CO-Hb concentration) and no residual is greater than 10.1.

In addition, a lasso regression was also performed on all available data (i.e. with exposure times between 1 and 100 ms and 40 cm and 60 cm objective distance). A total of 50 images were used. In this case, the RMSE was only 3.58 percentage points. Since in this case spectra recorded in the same week were included in both the training and test data, this result is less meaningful in terms of the predictive power of the model on new data. Nevertheless, this result shows that the prediction is robust to variables such as sample distance, ambient light, and camera settings. Furthermore, this result illustrates that the RMSE of the first regression model was not artificially improved by the selection of data.

## Discussion

The present study shows that hyperspectral imaging allows the CO-Hb concentration in human blood to be determined and qualitatively distinguished from a negative control sample by the contactless analysis of its spectrophotometric characteristics. The validity of the model created is limited by the idealized setup of the experiment in vitro and the limited amount of data for training the model.

Under standardized environmental conditions, the model achieved a prediction error of approx. 4.5 percentage points and maximum errors of less than 11 percentage points. Although this error varies overall within the six-week test, it does not increase linearly with the time elapsed, which is why we assume the method to remain constant over a longer period. If applicable in forensic medical practice, a distinction between pathologically elevated CO-Hb concentration (above 9—15% even in heavy smokers) [7] and physiological values could therefore be possible. This distinction would be even more precise for lethal CO intoxications, in which a concentration of between 30 and 60% CO-Hb is usually measured [13]. Furthermore, our method could fasten the time needed for evaluating COHb concentrations, since currently laboratories can take several days for their results, especially if they are not located near the body´s finding site or the coroner’s office as its often the case in rural areas.

For a practical application in post-mortem examinations (hyperspectral analysis of death spots, mucous membranes or nail beds), the scattering and absorption processes caused by melanin, collagen and water, for example, must also be taken into account, since they are some of the most important chromophores for visible wavelengths, of which the absorption coefficient decreases monotonically with the increasing wavelength [15, 19, 22, 23]. In a study conducted by Bohnert M. et al. [2], in which *livor mortis* were recorded with a spectrophotometer and analyzed with a stochastic “Monte Carlo” model, the experimental setup featured an immobile, complex recording device and an equally controlled environment. The calculation time of the model was more than 7 days and thus not superior to lab analyses timewise.

For our system, the training of the Lasso regression model only took a few seconds. With the help of the Specim IQ Studio® software, it would be possible to create evaluation procedures (algorithms, so-called "applications") for data processing and analysis and store them in the camera, so that the evaluation of the data takes place in the camera and the result is displayed to the examiner on site. In real blood samples from patients, the hemoglobin concentration and the potential presence of other hemoglobin derivatives—such as deoxyhemoglobin or methemoglobin—and their respective optical properties must also be considered for training of the model. For example, Principal Component Analysis (PCA), Neighborhood component feature selection (NCFS), and Support Vector Machines (SVM) have proofed to be capable of assigning typical spectral bands of individual hemoglobin derivates to spectral classes [3], which could make on-site analysis of the images possible within a few seconds and the result could then be visualized on the camera display.

A key limitation for those would involve situations where illumination conditions are either unstable or of low intensity. In such cases, the mobile light sources employed in this study could provide a uniform distribution of light at the scene. However, to address rapidly changing lighting conditions or extremely confined spaces with minimal movement range, a simple device could be developed. This device would function similarly to the commonly used “matte box” and incorporate a circular light source around the camera lens to ensure consistent illumination and offer some degree of shielding from ambient light interference.

The method presented could also give a second chance in complex cases of forensic investigations, where the integrity of samples yet to analyze has already been decreased by rot or heat-related circumstances. For us, this recently was the case with exhumed material (Postmortem Interval: 15 months) for an appraisal, in which spectrophotometric examination of the cadaveric blood was no longer possible. For the first time, the method developed here was applied to greatly damaged probes and was able to acquire spectrophotometric data, where standard measurement methods delivered only error messages, resulting in the model to at least classify the COHb concentration as “highly elevated “ and therefore provide an additional piece of information for the toxicological evaluation.

The described challenging forensic scenario demonstrates the method’s potential utility but also highlights that its applicability is limited once the level of destruction—such as coagulation, heat exposure, or the presence of burned organic matter—exceeds a certain threshold. The same would apply to skin samples, where burn wounds or charring would significantly compromise the method’s effectiveness.

## Summary

HSI, in combination with the regression model, enabled the rapid, non-contact, non-destructive, and mobile detection of carbon monoxide poisoning in artificially saturated blood from erythrocyte concentrates, with consistent results even after a six week storage of the samples. The setup, consisting of three tripods for mounting the camera and light sources, along with a table for the samples and reference card, is simple to assemble and potentially adaptable for use at any location.

Demonstrating the general feasibility, the method established here could thus extend the range of forensic applications of HSI techniques, while pre-analytical influencing factors (e.g. variable ambient light, scattering effects through tissue) must be considered before use in post-mortem examinations. Tissue-related scattering effects remain an area for future work and effects on the spectrophotometric approach from other hemoglobin derivatives will require further investigation with untreated blood samples containing them. Controlling ambient light in practical applications may still be realistic by designing attachments that shield external light and provide a controlled light source.

## Data Availability

The datasets generated during and/or analyzed during the current study are available from the corresponding author on reasonable request.

## Code availability

The code generated during the current study are available from the corresponding author on reasonable request.
